# Clinical significance of WES-defined *ERBB2* amplification in resectable colorectal cancer

**DOI:** 10.1007/s10147-026-03161-5

**Published:** 2026-08-20

**Authors:** Kazuki Kishi, Atsushi Hamabe, Yusuke Suwa, Naoya Akazawa, Keiji Hirata, Masataka Ikeda, Mitsuru Yokota, Kentaro Kato, Masahito Kotaka, Yujiro Nishizawa, Hideaki Bando, Yoshiaki Nakamura, Saori Mishima, Tadayoshi Hashimoto, Arkarachai Fungtammasan, Charuta C. Palsuledesai, Robert W. Lentz, Daisuke Kotani, Hiroya Taniguchi, Jun Watanabe, Ichiro Takemasa, Takeshi Kato, Mamoru Uemura, Hidetoshi Eguchi, Yuichiro Doki, Eiji Oki, Takayuki Yoshino

**Affiliations:** 1 Department of Gastroenterological Surgery, Graduate School of Medicine, The University of Osaka, Suita, Osaka Japan; 2 Department of Surgery, Gastroenterological Center, Yokohama City University Medical Center, Yokohama, Kanagawa Japan; 3 Department of Gastroenterological Surgery, Sendai City Medical Center (Sendai Open Hospital), Sendai, Miyagi Japan; 4 Department of Surgery, School of Medicine, University of Occupational and Environmental Health, Kitakyushu, Fukuoka Japan; 5 Division of Colorectal Surgery, Department of Gastrointestinal Surgery, Hyogo Medical University, Hyogo, Japan; 6 Department of General Surgery, Kurashiki Central Hospital, Kurashiki, Okayama Japan; 7 Department of Surgery, Teine-Keijinkai Hospital, Sapporo, Hokkaido Japan; 8 Gastrointestinal Cancer Center, Sano Hospital, Kobe, Hyogo Japan; 9 Department of Gastroenterological Surgery, Osaka General Medical Center, Sumiyoshi-ku, Osaka, Japan; 10 Department of Gastrointestinal Oncology, National Cancer Center Hospital East, Kashiwa, Chiba Japan; 11 Natera, Inc, Austin, TX USA; 12 Department of Clinical Oncology, Aichi Cancer Center Hospital, Nagoya, Aichi Japan; 13 Department of Colorectal Surgery, Kansai Medical University, Hirakata, Osaka Japan; 14 Department of Gastroenterological Surgery, Osaka International Medical and Science Center, Osaka City, Osaka Japan; 15 NHO Osaka National Hospital, Chuo-ku, Osaka, Japan; 16 Department of Advanced Medicine and Innovative Technology, Kyushu University, Higashi-ku, Fukuoka, Japan

**Keywords:** HER2 amplification, Resectable colorectal cancer, Prognostic marker, Relevance to adjuvant chemotherapy efficacy, ctDNA

## Abstract

**Background:**

*HER2* amplification is demonstrated to influence response to therapy and outcomes in metastatic colorectal cancer (mCRC), but its role in resectable CRC remains unclear. This study investigated the prognostic impact of WES-defined *ERBB2* amplification and its association with adjuvant chemotherapy (ACT) efficacy and circulating tumor DNA (ctDNA) in resectable CRC.

**Methods:**

Data from 3607 patients with resectable CRC from the GALAXY study were analyzed. *HER2* amplification was defined using *ERBB2* copy number alterations based on whole exome sequencing of tumor specimens. We analyzed disease-free survival (DFS) in WES-defined *ERBB2* amplified CRC, evaluating ACT benefit and the correlation between *ERBB2* copy number and ctDNA status.

**Results:**

No significant difference in DFS was observed between those with and without WES-defined *ERBB2* amplification in a univariate (*P* = 0.0659) or a multivariate analysis (*P* = 0.2300). Among patients with pathological stage III, ACT was associated with improved DFS in the non-amplified subgroup (HR 0.67, *P* = 0.0002), particularly in the subgroup of patients with early post-operative (2–10 weeks following surgical resection) ctDNA-positivity (HR 0.27, *P* < 0.0001), whereas no clear association was detected in the small amplified subgroup; however, the ACT-by-*ERBB2* amplification interaction was not statistically significant. While WES-defined *ERBB2* amplification correlated with higher early post-operative ctDNA positivity (*P* = 0.0486), *ERBB2* copy number did not associate with recurrence or levels. However, early post-operative ctDNA positivity strongly predicted poor DFS in patients with WES-defined *ERBB2* amplification (HR 15.26, *P* < 0.0001).

**Conclusions:**

WES-defined *ERBB2* amplification was not identified as a significant adverse prognostic factor in resectable CRC, unlike early post-operative ctDNA status. The limited number of amplified cases precluded a reliable assessment of whether WES-defined *ERBB2* amplification modifies the association between ACT and DFS.

**Supplementary Information:**

The online version contains supplementary material available at https://doi.org/10.1007/s10147-026-03161-5.

## Introduction

Amplification and/or overexpression of human epidermal growth factor receptor 2 (*HER2*) gene, referred to as HER2 positivity, is relatively uncommon in colorectal cancer (CRC), with an estimated prevalence of approximately 2–4% [1, 2]. Data from population-based and trial cohorts report HER2 positivity in approximately 2–5% of metastatic CRC (mCRC), compared with ~ 1–2% of resected stage II–III tumors [3–5]. In mCRC, numerous studies have demonstrated that HER2 positivity influences response to therapy and clinical outcomes. In particular, patients with HER2-positive, wild-type *RAS* mCRC show poor response to anti-EGFR therapy and shorter progression-free survival (PFS) [6, 7]. HER2 positivity has also been associated with unfavorable overall survival (OS) in mCRC [7]. Recently, HER2-targeted therapy has emerged as a promising treatment strategy for mCRC. The efficacy of dual HER2 blockade with trastuzumab-based regimens has been confirmed in multiple clinical trials [8–12]. In Japan, pertuzumab plus trastuzumab was approved in 2022 for HER2-positive mCRC, and in 2023, the combination of tucatinib and trastuzumab received regulatory approval. These advances have firmly established HER2 as an important therapeutic target in mCRC.

In contrast, the clinical significance of *HER2* amplification/overexpression in resectable CRC remains unclear. Although several studies have suggested its associations with postoperative recurrence, prognosis, and the efficacy of adjuvant chemotherapy (ACT) [13–15], evidence is still insufficient to determine whether HER2 positivity serves as an independent prognostic or predictive factor in this setting. This uncertainty likely reflects both its low prevalence and the fact that HER2 testing is not routinely performed in stage I–III CRC in clinical practice.

With recent advances in next-generation sequencing (NGS), genetic alterations and copy number aberrations—including *KRAS* mutations and *HER2* amplification—can now be detected within a single assay. Furthermore, assessment of *HER2* amplification in CRC using NGS-based assays has shown high concordance with immunohistochemistry (IHC) and fluorescence in situ hybridization (FISH) results [16], supporting its reliability for detecting *HER2* amplification. These advances provide an opportunity to systematically evaluate the clinical relevance of WES-defined *ERBB2* amplification in large cohorts of patients with resectable CRC.

The GALAXY study, which is the observational arm of the ongoing CIRCULATE-Japan study (UMIN000039205), enrolled more than 6000 patients with resectable, clinical stage (cStage) II-IV CRC. Whole-exome sequencing (WES) of tumor tissues was performed for each patient to design personalized, tumor-informed circulating tumor DNA (ctDNA) assays for evaluating recurrence risk. In this study, we leveraged this large and homogeneous data from the GALAXY study and evaluated the clinical significance of WES-defined *ERBB2* amplification in resectable CRC.

## Methods

### Patients and study design

This study is a secondary analysis using data from the GALAXY study, the observational arm of the prospective, multicenter CIRCULATE-Japan study. The GALAXY study is a nationwide registry that prospectively monitors ctDNA status after complete surgical resection in patients with cStage II-IV CRC [17, 18]. It serves to screen patients for ctDNA-based molecular residual disease (MRD) status and subsequently assign patients to one of the two randomized ctDNA-guided interventional phase 3 trials: ALTAIR (treatment escalation in MRD-positive patients) and VEGA (treatment de-escalation in MRD-negative patients) [17, 19]. The detailed study protocol has been previously reported [19]. In addition, the eligibility and exclusion criteria, procedures for sample collection and testing, administration of ACT, definition of DFS, and methods for recurrence assessment have been described in the interim analyses [17, 18]. A total of 6,061 patients were enrolled between May 8, 2020, and March 31, 2024.

**Fig. 1:**
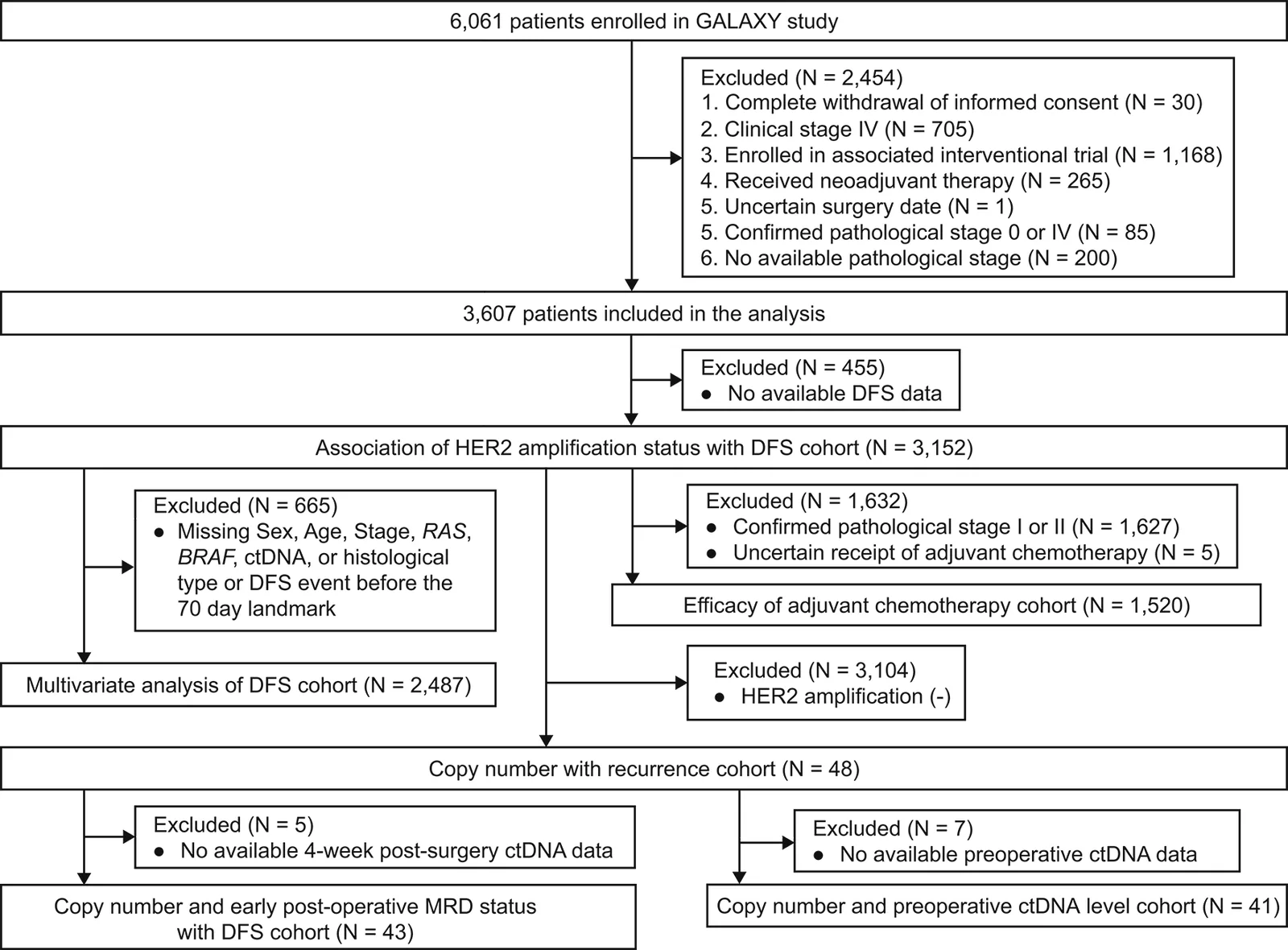
CONSORT diagram illustrating the inclusion of patients

For this current study, we excluded those who withdrew consent, received neoadjuvant therapy, had an uncertain date of surgery, or enrolled in the interventional ALTAIR or VEGA studies (Fig. 1). This resulted in a cohort of 3,607 patients with cStage II-III and pathologic stage (pStage) I-III CRC for this analysis. In each subgroup analysis, patients with missing data for the analyzed variables were excluded. No patients in this analysis received anti-EGFR or anti-HER2 therapies.

### Personalized, tumor-informed ctDNA analysis

Early post-operative ctDNA-based MRD status during 2–10 weeks following surgical resection was assessed as part of the GALAXY study using a clinically validated, personalized, tumor-informed 16-plex mPCR-NGS assay (Signatera™, Natera, Inc.), as previously described [20–22]. Briefly, a set of up to 16 single nucleotide variants (SNVs) was identified from paired WES data generated from fixed-formalin paraffin-embedded (FFPE) tumor tissue and normal blood samples. These SNVs were tracked in the associated patient’s plasma using a multiplex PCR-based NGS approach. Samples with at least 2 patient-specific SNVs detected above a predefined threshold were considered ctDNA-positive (MRD-positive). ctDNA concentrations (levels) were reported as mean tumor molecules per mL of plasma (MTM/mL).

### Definition of WES-based ERBB2 amplification

*ERBB2* copy number was estimated from WES-based log2 fold-change values using the following formula: CN = 2 × 2^(log2FC)^. The cut-off for *ERBB2* amplification as defined by copy number varies between 3 and 8 [23]. In the WES analysis of our cohort, the lowest *ERBB2* copy number among tumors showing copy-number gain was 4.59.　Based on this WES-defined *ERBB2* amplification was defined as a *ERBB2* copy number greater than 4.0, based on WES analysis of tumor specimens. We were unable to evaluate tumor purity, ploidy, or the focality of amplification in the current dataset.

### Statistical analysis

Categorical variables were compared using the chi-square or Fisher’s exact test, and continuous variables using the Mann–Whitney U test. Survival analyses were conducted in R version 4.4.3 with the survival (version 3.8.3) and survminer (version 0.5.0) packages. Survival curves were estimated by the Kaplan–Meier method, group differences evaluated with the log-rank test, and hazard ratios calculated using Cox proportional hazards models and Firth corrected-Cox from package coxphf (version 1.13.4) was used when event count is low. Multivariate Cox models were used to identify prognostic factors associated with DFS. Box plots were generated with ggplot2 (version 3.5.2) and ggpubr (version 0.6.1), and data manipulation performed with dplyr (version 1.1.4). *P* values < 0.05 were considered statistically significant.

A landmark analysis, which offset the disease-free survival (DFS) starting point by 70 days, was performed using Kaplan-Meier survival estimates and a Multivariate Cox proportional hazards model incorporating early post-operative MRD status. This 70 days offset was implemented to prevent immortal time bias (guarantee-time bias). By applying this landmark, we ensured that patients who experienced rapid recurrence or death prior to the completion of the standard post-operative MRD testing window were appropriately excluded, allowing for an unbiased comparison between the MRD-positive and MRD-negative cohorts. The number of patients and DFS events excluded before the landmark is reported in Supplementary Table 1.

The sensitivity analysis was performed for association of WES-defined *ERBB2* amplification with DFS in univariate and multivariate analysis, and efficacy of ACT based on WES-defined *ERBB2* amplification status using the ERBB2 copy number between 3 and 20.

## Results

### Patient characteristics

The baseline patient and tumor characteristics are shown in Table 1. Among 3,607 patients, 59 (1.6%) had WES-defined *ERBB2* amplified tumors, i.e. *ERBB2* copy number greater than 4.0. We noted that the cutoff at *ERBB2* copy number 3 or 4 would give the same results in this cohort (Fig. S1). The WES-defined *ERBB2* amplified group included a higher proportion of pStage III cases (61.0% vs. 47.1%; *P* = 0.0358) and lower frequencies of *RAS* mutations (8.5% vs. 38.4%; *P* < 0.0001), *BRAF* V600E mutation (0% vs. 8.4%; *P* = 0.0083), and MSI-high status (0% vs. 11.1%; *P* = 0.0014). Interestingly, early post-operative MRD positivity during 2–10 weeks post-surgery was higher in the WES-defined *ERBB2* amplified group (18.6% vs. 9.9%; *P* = 0.0486).

**Table 1:** Baseline demographic and clinical characteristics of patients with and without WES-defined *ERBB*2 amplification

Characteristic		WES-defined ERBB2 amp (−) (*N* = 3548) 98.4%	WES-defined ERBB2 amp (+) (*N* = 59) 1.6%	*P* value (Fisher or Wilcoxon)
Sex	Male	1844 (52.0%)	33 (55.9%)	0.6002
	Female	1704 (48.0%)	26 (44.1%)	
Age, years	median (IQR)	69 (59–75)	66 (58–73)	0.3816
Primary tumor location	Right (C-T)	1260 (35.5%)	14 (23.7%)	0.1170
	Left (D-RS)	1724 (48.6%)	32 (54.2%)	
	Rectum (Ra, Rb)	564 (15.9%)	13 (22.0%)	
Histological type	Differentiated	3362 (94.8%)	58 (98.3%)	0.2628
	Poorly differentiated/ Signet-ring cell	115 (3.2%)	0 (0%)	
	Other/Unknown	71 (2.0%)	1 (1.7%)	
Pathologic stage	Stage I	675 (19.0%)	7 (12%)	0.0358
	Stage II	1202 (33.9%)	16 (27%)	
	Stage III	1671 (47.1%)	36 (61%)	
*RAS* mutations	Wild type	1919 (54.1%)	52 (88.1%)	< 0.0001
	Mutant	1364 (38.4%)	5 (8.5%)	
	Unknown	265 (7.5%)	2 (3.4%)	
*BRAF* V600E mutation	No	2982 (84.0%)	57 (96.6%)	0.0083
	Yes	297 (8.4%)	0 (0%)	
	Unknown	269 (7.6%)	2 (3.4%)	
MSI	MSI-high	393 (11.1%)	0 (0%)	0.0014
	MSS	2828 (79.7%)	57 (96.6%)	
	Unknown	327 (9.2%)	2 (3.4%)	
Early post-operative MRD status (2–10 weeks post-surgery)	Negative	2782 (78.4%)	43 (72.9%)	0.0486
	Positive	352 (9.9%)	11 (18.6%)	
	Unknown	414 (11.7%)	5 (8.5%)	

*IQR* Interquartile range; Right (C-T), cecum to transverse colon (proximal colon); Left (D-RS), descending colon to Rectosigmoid (distal colon); Ra, upper rectum; Rb, lower rectum; MSI, microsatellite instability; MSS, microsatellite stable; MRD, molecular residual disease. *P *value was calculated using Fisher’s exact test among non-missing categories

### Association of WES-defined ERBB2 amplification status with DFS

**Fig. 2:**
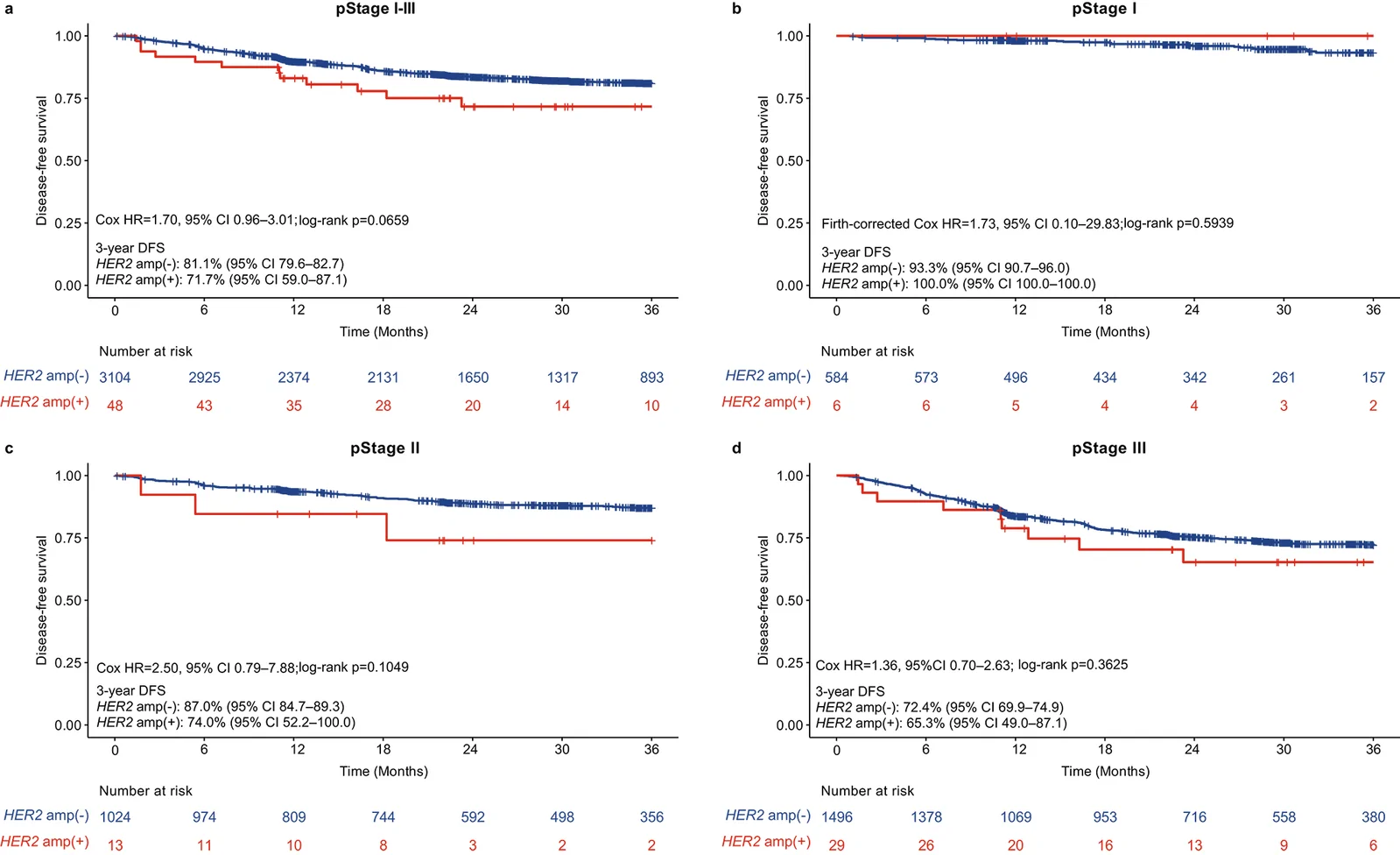
Association of WES-defined ERBB2 amplification with DFS. Kaplan–Meier estimates of DFS stratified by WES-defined ERBB2 amplification status among all patients with available DFS data (*N* = 3152) (**a**), patients with pStage I (*N* = 590) (**b**), pStage II (*N* = 1037) (**c**), or pStage III (*N* = 1525) (**d**) disease. DFS, disease-free survival; pStage, pathological stage

Among 3152 patients with available DFS data, the median follow-up was 783 days (IQR: 372–1107) as of March 12, 2025. Recurrence or death occurred in 12 of 48 patients with WES-defined *ERBB2* amplification (25.0%) and in 520 of 3,104 patients without amplification (16.8%). Comparison of recurrence patterns between the WES-defined *ERBB2* amplified and non-amplified groups showed no remarkable differences in the sites of recurrence (Supplementary Table 2). The 3 year DFS was 71.7% in patients with WES-defined *ERBB2* amplification and 81.1% in patients without amplification, but the difference was not statistically significant (hazard ratio [HR]: 1.70; 95% confidence interval [CI]: 0.96–3.01; *P* = 0.0659) (Fig. 2a). Similarly, analyses stratified by pStage showed no significant association between WES-defined *ERBB2* amplification and DFS (Fig. 2b–d).

**Fig. 3:**
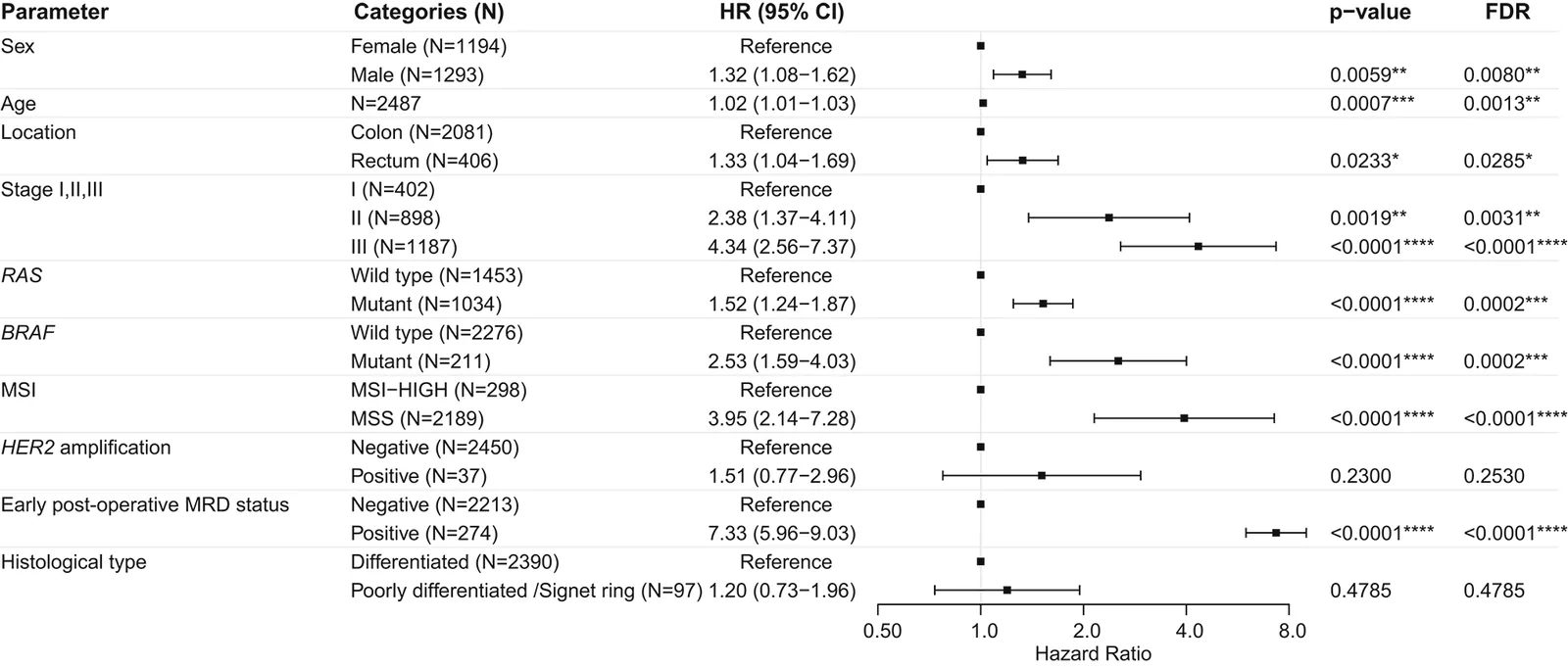
Multivariable analysis of DFS among patients with complete clinicopathologic data available (*N* = 2487). Landmark analysis was performed

To further assess the prognostic significance of WES-defined *ERBB2* amplification while accounting for other clinicopathological factors, a multivariable analysis was conducted in 2487 patients with complete data and who were event-free until the 70 days landmark. In the multivariate analysis, WES-defined *ERBB2* amplification was not observed to be a significant adverse prognostic factor (HR: 1.51; 95% CI: 0.77–2.96; *P* = 0.2300; Fig. 3), whereas early post-operative MRD positivity was the strongest adverse factor of DFS (HR: 7.33; 95% CI: 5.96–9.03; *P* < 0.0001; Fig. 3). Other statistically significant adverse prognostic factors included advanced pStage (II vs. I: HR: 2.38; 95% CI: 1.37 − 4.11; *P* = 0.0019; III vs. I: HR: 4.34, 95% CI: 2.56 − 7.37, *P* < 0.0001), MSS (HR: 3.95; 95% CI: 2.14–7.28; *P* < 0.0001), BRAF V600E mutation (HR: 2.53; 95% CI: 1.59–4.03; *P* < 0.0001), RAS mutations (HR: 1.52; 95% CI: 1.24–1.87; *P* < 0.0001), male sex (HR: 1.32; 95% CI: 1.08–1.62; *P* = 0.0059), and rectal location (HR: 1.33; 95% CI: 1.04–1.69; *P* = 0.0233) (Fig. 3). Considering that adjustment for early post-operative MRD status may affect the interpretation of the prognostic value of WES-defined *ERBB2* amplification, multivariable analysis excluding MRD status was also conducted; however, WES-defined *ERBB2* amplification was not significantly associated with DFS in the model. (Supplementary Fig. 2).

### Efficacy of ACT stratified by WES-defined ERBB2 amplification and MRD status

Because ACT is recommended as the standard of care for pStage III CRC, we analyzed its benefit in that patient subgroup upon stratifying by WES-defined *ERBB2* amplification status. The baseline characteristics according to ACT receipt among pStage III patients are shown in Table 2 for the overall cohort and in Supplementary Tables 3 and 4 for cohorts stratified by WES-defined *ERBB2* amplification status. Age and early post-operative MRD status within 2–10 weeks post-surgery differed significantly between the ACT-treated and untreated groups in the overall cohort and in the non-amplified subgroup. However, no statistically significant differences in baseline characteristics were observed in the WES-defined *ERBB2* amplified subgroup. The distribution of ACT regimens and treatment durations are shown in Supplementary Tables 5 and 6.

**Table 2:** Baseline demographic and clinical characteristics of pStage III patients stratified by ACT receipt

Characteristic		ACT No (*n* = 428)	ACT Yes (*n* = 1092)	*P* value (Fisher or Wilcoxon)
Sex	Male	233 (54.4%)	572 (52.4%)	0.4931
	Female	195 (45.6%)	520 (47.6%)	
Age, years	Median (IQR)	71 (62–77)	66 (56–73)	< 0.0001
Primary tumor location	Right (C-T)	145 (33.9%)	366 (33.5%)	0.6755
	Left (D-RS)	215 (50.2%)	532 (48.7%)	
	Rectum (Ra, Rb)	68 (15.9%)	194 (17.8%)	
Histological type	Differentiated	410 (95.8%)	1014 (92.9%)	0.3993
	Poorly differentiated/ Signet-ring cell	15 (3.5%)	50 (4.6%)	
	Other/Unknown	3 (0.7%)	28 (2.6%)	
WES-defined *ERBB2* amplification	Negative	421 (98.4%)	1070 (98.0%)	0.8350
	Positive	7 (1.6%)	22 (2.0%)	
*RAS* mutations	Wild type	241 (56.3%)	596 (54.6%)	0.5201
	Mutant	173 (40.4%)	463 (42.4%)	
	Unknown	14 (3.3%)	33 (3.0%)	
*BRAF* V600E mutation	No	380 (88.8%)	967 (88.6%)	1.0000
	Yes	34 (7.9%)	89 (8.2%)	
	Unknown	14 (3.3%)	36 (3.3%)	
MSI	MSI-high	39 (9.1%)	75 (6.9%)	0.1608
	MSS	373 (87.1%)	960 (87.9%)	
	Unknown	16 (3.7%)	57 (5.2%)	
Early post-operative MRD status (2–10 weeks post-surgery)	Negative	337 (78.7%)	696 (63.7%)	0.0059
	Positive	59 (13.8%)	191 (17.5%)	
	Unknown	32 (7.5%)	205 (18.8%)	

IQR, interquartile range; Right (C-T), cecum to transverse colon (proximal colon); Left (D-RS), descending colon to Rectosigmoid (distal colon); Ra, upper rectum; Rb, lower rectum; MSI, microsatellite instability; MSS, microsatellite stable; MRD, molecular residual disease. *P *value was calculated using Fisher’s exact test among non-missing categories

**Fig. 4:**
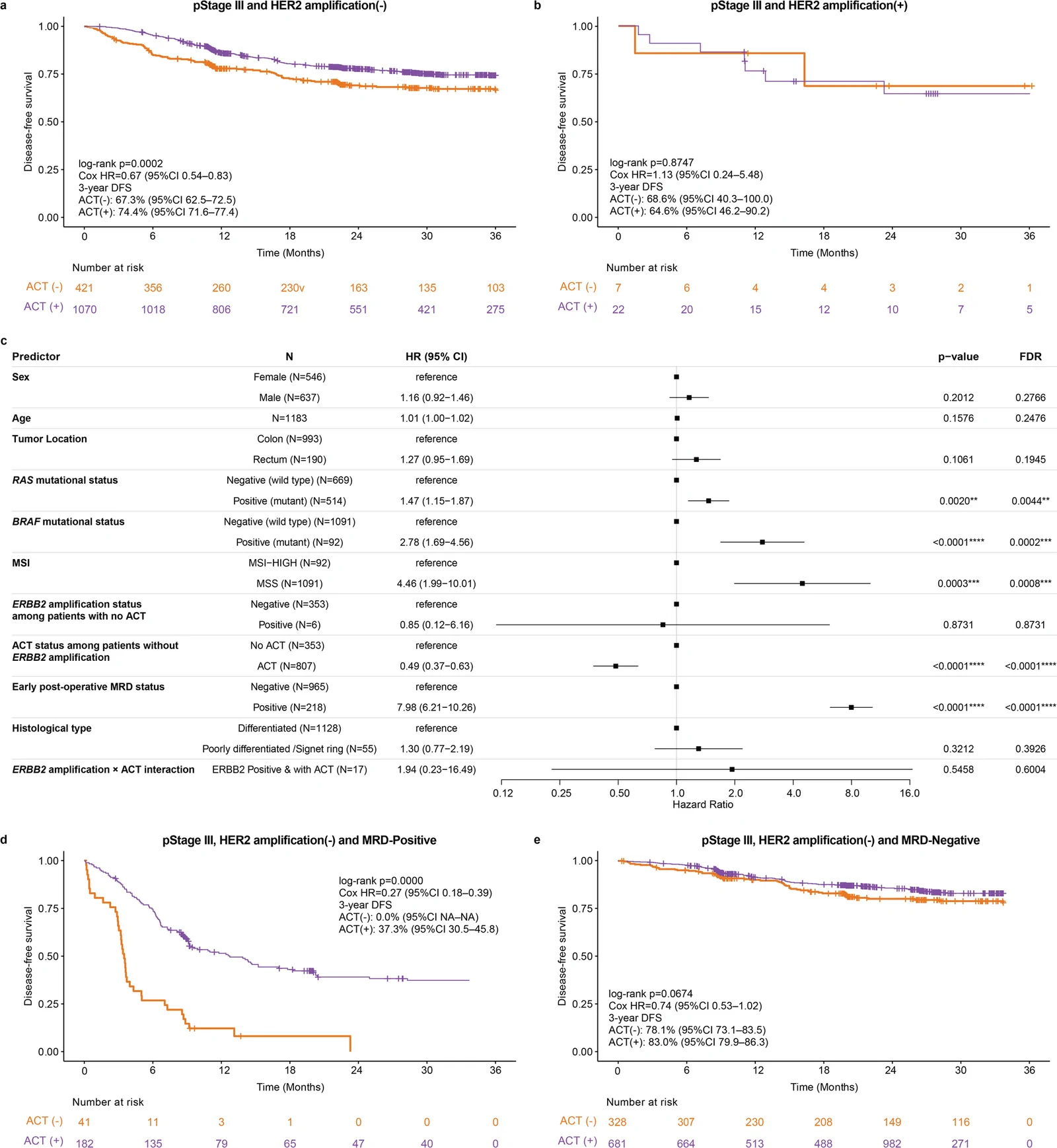
Efficacy of ACT based on WES-defined ERBB2 amplification status.**a**, **b** Kaplan–Meier estimates of DFS stratified by ACT status among pStage III patients without WES-defined ERBB2 amplification (*N* = 1491) (**a**) and with WES-defined ERBB2 amplification (*N* = 29) (**b**). **c** Multivariable analysis of DFS including an ACT × WES-defined ERBB2 amplification interaction term among pStage III patients with complete clinicopathologic data (*N* = 1183). **d**, **e** Kaplan–Meier estimates of DFS stratified by ACT status in pStage III patients without WES-defined ERBB2 amplification and with early post-operative MRD-positivity (*N* = 223) (**d**), or early post-operative MRD-negativity (*N* = 1009) (**e**). Landmark analysis was performed for panels c-e. Analyses stratified by MRD status and ACT status among patients with WES-defined ERBB2 amplification are not included due to small sample size. DFS, disease-free survival; pStage, pathological stage; ACT, adjuvant chemotherapy

Among patients without WES-defined *ERBB2* amplification, ACT conferred a survival benefit (HR: 0.67; 95% CI: 0.54–0.83; *P* = 0.0002), with a 3 year DFS rate of 74.4% among those receiving ACT versus 67.3% in those without ACT (Fig. 4a). In contrast, no significant benefit from ACT was observed among patients with WES-defined *ERBB2* amplification (HR: 1.13; 95% CI: 0.24–5.48; *P* = 0.8747), with 3 year DFS rates of 64.6% and 68.6%, respectively (Fig. 4b). To evaluate whether the association between ACT and DFS differed according to WES-defined *ERBB2* amplification status after adjustment for other clinicopathological factors, we performed an interaction analysis including an ACT × WES-defined *ERBB2* amplification interaction term (Fig. 4c). The interaction was not statistically significant (interaction HR: 1.94, 95% CI: 0.23–16.49, *P* = 0.5458).

Given that early postoperative MRD status is previously shown to be strongly predictive of benefit of ACT, we further analyzed the efficacy of ACT among patients stratified by MRD status within 2–10 weeks post-surgery. Among patients without WES-defined *ERBB2* amplification, those with MRD-positivity derived significant benefit from ACT (HR: 0.27, 95% CI: 0.18–0.39, *P* < 0.0001; 3 year DFS: 37.3% for ACT group vs. 0% for no ACT group; Fig. 4d). In contrast, no statistically significant benefit of ACT was observed among patients without WES-defined *ERBB2* amplification who were MRD -negative (HR: 0.74, 95% CI: 0.53–1.02, *P* = 0.0674; 3 year DFS: 83.0% for ACT group vs. 78.1% for no ACT group; Fig. 4e). Analyses stratified by MRD and ACT status could not be performed among patients with WES-defined *ERBB2* amplification due to low sample size.

In the WES-defined *ERBB2* non-amplified subgroup, multivariable Cox analyses demonstrated a significant association between ACT and improved DFS, including in an additional model adjusted for age and early post-operative MRD status (Supplementary Fig. 3a, b). In contrast, in the WES-defined *ERBB2* amplified subgroup, ACT was not significantly associated with improved DFS in a multivariable model adjusted for age and early post-operative MRD status (Supplementary Fig. 3c).

### Prognostic value of ERBB2 copy number and MRD status in patients with WES-defined ERBB2 amplification

**Fig. 5:**
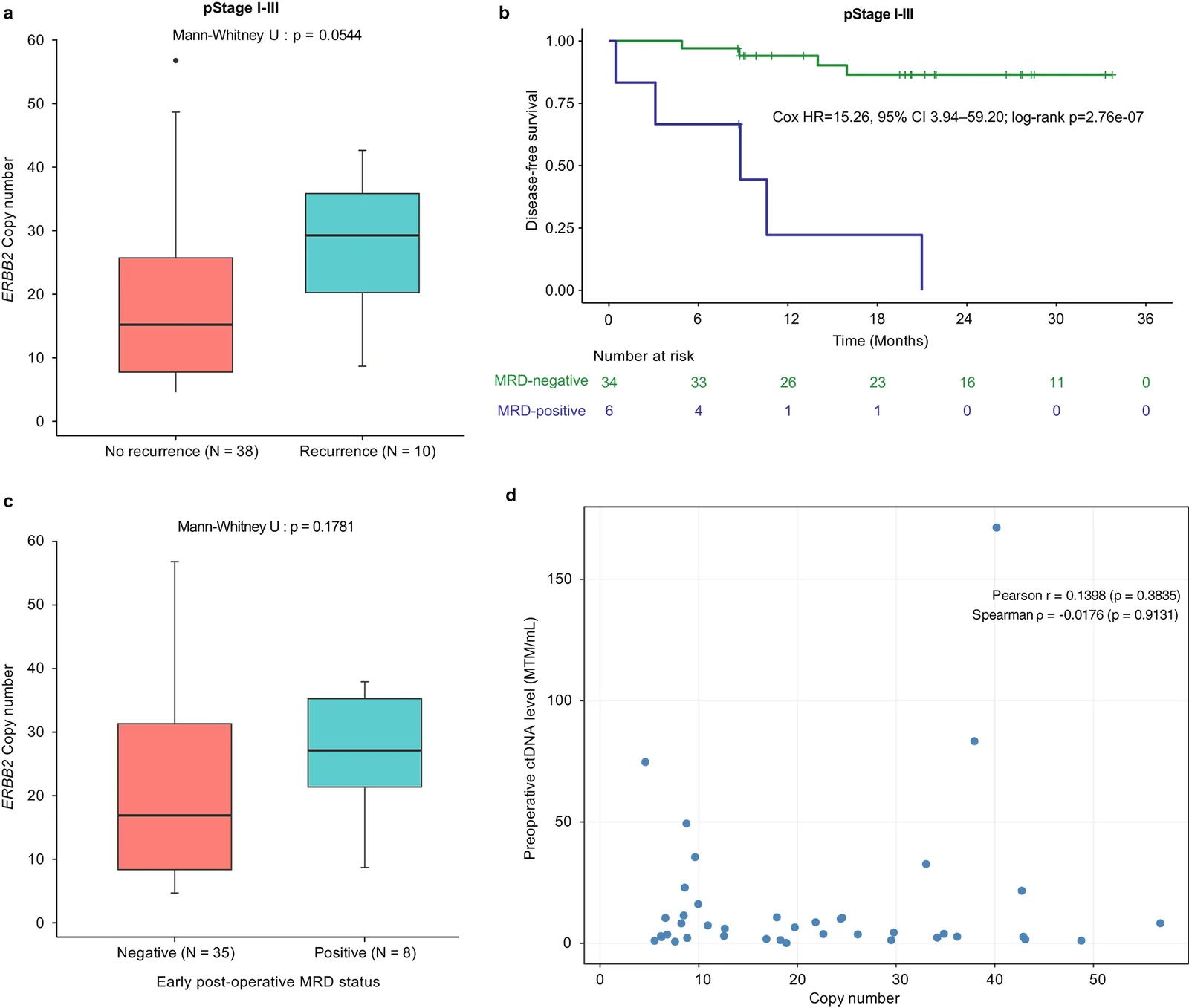
Prognostic significance and association between*ERBB2*copy number and early post-operative MRD status (2–10 weeks post-surgery).**a** ERBB2 copy number stratified by recurrence status in patients with WES-defined ERBB2 amplification and pStages I-III CRC (*N* = 48). **b** Kaplan–Meier curve for DFS stratified by early post-operative MRD status in patients with WES-defined ERBB2 amplification and pStages I-III CRC (*N* = 40). **c***ERBB2* copy number in patients with WES-defined *ERBB2* amplification, stratified by early post-operative MRD status (*N* = 43). **d** Scatter plot of *ERBB2* copy number and ctDNA levels (MTM/mL) at pre-operative (*N* = 41). Landmark analysis was performed for survival analyses in panel b. DFS, Disease-free survival

Next, we examined the prognostic significance of *ERBB2* copy number and early post-operative MRD status among patients with WES-defined *ERBB2* amplified resectable CRC. Patients who later developed a recurrence showed a trend towards higher *ERBB2* copy numbers compared to those who remained recurrence-free, although the difference was not statistically significant (median copy number: 29.27 [IQR, 20.26–35.85] vs. 15.22 [IQR, 7.76–25.73], *P* = 0.0544) (Fig. 5a). In contrast, early post-operative MRD positivity was strongly associated with poor DFS (HR: 15.26, 95% CI: 3.94–59.20, *P* < 0.0001) (Fig. 5b). When analysis was limited to patients with pStage III disease, similar trends were observed with recurrent cases having higher *ERBB2* copy numbers without statistical significance (Median Copy Number: 28.00 [IQR, 17.53–35.19] vs. 13.78 [IQR, 8.24–29.76], *P* = 0.2373), whereas MRD-positive patients exhibited significantly shorter DFS (HR: 9.15, 95% CI: 1.98–42.321, *P* = 0.0007) (Supplementary Fig. 4).

Finally, *ERBB2* copy number did not significantly differ between patients who were MRD-positive or MRD-negative early post-operatively (median copy number : 27.07 [IQR, 21.32–35.19] vs. 16.86 [IQR, 8.36–31.27], *P* = 0.1781) (Fig. 5c) and showed no correlation with preoperative ctDNA levels (Pearson *r* = 0.1398, *P* = 0.3835; Spearman ρ = − 0.0176, *P* = 0.9131) (Fig. 5d).

### Sensitivity analyses

Sensitivity analyses were conducted to assess whether the main findings were robust to changes in the definition of WES-defined *ERBB2* amplification. The univariate association of WES-defined *ERBB2* amplification with DFS showed an inconsistent trend below and above the *p *value 0.05 (Supplementary Fig. 5a). On the other hand, the multivariate analysis consistently showed WES-defined *ERBB2* amplification to have no significant association with DFS at all cutoff values tested (Supplementary Fig. 5b).

In analyses evaluating efficacy of ACT among pStage III patients, all analyses remained consistent with those described above after changing the cutoff (Supplementary Fig. 5c–f).

## Discussion

Given the low prevalence and limited existing literature, the clinical significance of *HER2* amplification in resectable CRC has remained poorly defined. Leveraging WES data from the GALAXY study, this large-scale analysis characterized the clinicopathologic features of *HER2*-amplified resectable CRC, a rare but biologically distinct molecular subset. Our findings suggest that while WES-defined *ERBB2* amplification is not significantly prognostic of DFS outcomes, a modest adverse prognostic effect cannot be excluded. Additionally, we found that early postoperative MRD positivity is a significant adverse prognostic factor in WES-defined *ERBB2* amplified resectable CRC. Although the univariate analyses indicated a trend for association between WES-defined *ERBB2* amplification and poorer DFS, its prognostic impact as a significant, independent adverse factor was not observed in the multivariate analysis. Recent studies have also reported that high HER2 expression is associated with poor prognosis in resectable CRC [13, 14]. However, a previous study reported no significant association between *HER2* amplification and recurrences in resectable CRC [24], and the prognostic significance of *HER2* amplification in resectable CRC therefore remains controversial and warrants further investigation.

We also examined whether WES-defined *ERBB2* amplification influences responsiveness to ACT among patients with pStage III disease. Patients without WES-defined *ERBB2* amplification derived a clear benefit from ACT. Interestingly, in this group without WES-defined *ERBB2* amplification, early post-operative MRD -positivity (2–10 weeks post-surgery) further identified a subpopulation likely to benefit from ACT, whereas patients with MRD-negativity derived no significant benefit from ACT. In contrast, a clear benefit from ACT was not detected among patients with WES-defined *ERBB2* amplification. However, because the ACT-by-*ERBB2* amplification interaction was not statistically significant, the present data did not provide evidence that WES-defined *ERBB2* amplification modifies the association between ACT and DFS. The limited number of amplified cases precluded a reliable assessment of whether WES-defined *ERBB2* amplification modifies the association between ACT and DFS and validation in larger cohorts is required. Although HER2-mediated activation of the PI3K/AKT pathway is a plausible mechanism, as dysregulation of this pathway has been implicated in resistance to both 5-fluorouracil and oxaliplatin [25–30], our data did not allow conclusions regarding whether this mechanism influences ACT responsiveness in resectable CRC. The efficacy of HER2-targeted therapies is reported in mCRC [8–12] and ACT combined with HER2-targeted therapy is established as the standard of care in resectable HER2-positive breast cancer. Given the biological rationale for HER2 signaling and the uncertainty of our findings, larger prospective studies are warranted to evaluate whether HER2 amplification influences the association between ACT and DFS and whether HER2-targeted strategies have a role in the adjuvant setting for resectable CRC.

In this study, the absolute *ERBB2* copy number was not significantly associated with recurrence or with early postoperative ctDNA-based MRD positivity and preoperative ctDNA levels. However, we found that early post-operative MRD positivity was a strong predictor of recurrence in WES-defined *ERBB2* amplified cases, even upon limiting the analysis to pStage III disease. These results suggest that postoperative MRD status can aid recurrence risk stratification in WES-defined *ERBB2* amplified CRC and may help identify candidates for adjuvant HER2-targeted therapy in future studies.

A strength of this study is the uniform WES-based assessment of *ERBB2* copy number across all samples. *HER2* amplification was defined with an *ERBB2* copy number threshold of > 4.0 based on WES data. Nearly all (96%, 57/59) of WES-defined *ERBB2* amplified tumors exceeded an *ERBB2* copy number of > 5.0, a previously published threshold for *HER2* positivity by IHC/FISH in mCRC [31]. Additionally, our observed frequency of WES-defined *ERBB2* amplification (1.6%) and clinicopathological features of our cohort, such as higher *HER2* amplification rates in pStage III, *RAS*/*BRAF* wild-type, and MSS tumors, are consistent with previous reports in resectable CRC [1, 5, 13, 14].

This study has several limitations. Its retrospective design introduces potential biases related to missing or incomplete data. Despite the large overall cohort, the small number of WES-defined *ERBB2* amplified cases, limited number of events, and the relatively limited follow-up with evaluation restricted to 3 year DFS may have reduced statistical power. Therefore, the therapeutic implications of our findings should be interpreted cautiously and validated in larger datasets.

## Conclusions

This study suggested that WES-defined *ERBB2* amplification is not a significant adverse prognostic factor in resectable CRC after accounting for ctDNA-based MRD status and other clinicopathological variables and did not provide evidence that WES-defined *ERBB2* amplification modifies the association between ACT and DFS. The limited number of amplified cases precluded a reliable assessment of whether WES-defined *ERBB2* amplification modifies the association between ACT and DFS. To our knowledge, this is the largest study to date addressing WES-defined *ERBB2* amplification as a prognostic and predictive biomarker in resected CRC. Further validation in larger-scale studies is warranted.

## Supplementary Information

Below is the link to the electronic supplementary material.

Supplementary Material 1

## Data Availability

Clinical and sequencing data from the GALAXY study are available under restricted access due to ethical and legal constraints. For detailed data sharing policies, please refer to the prior publications [17, 18]. Any requests will be reviewed within a time frame of 2 to 3 weeks by the CIRCULATE-Japan study steering committee to verify whether the request is subject to any intellectual property or confidentiality obligations. All data shared will be de-identified.

## Code availability

The fully documented code to generate raw outputs from the R statistical computing environment for analyses related to this manuscript are deposited at the GitHub repository and can be accessed at https://github.com/Natera-TMED/Kishi-et-al_HER2_Galaxy.
