# Supplementary material for: Clinical significance of WES-defined ERBB2 amplification in resectable colorectal cancer
Source: Int J Clin Oncol. 2026 Aug 20;31(10):2512–23. doi: 10.1007/s10147-026-03161-5 (PMC13615122; doi:10.1007/s10147-026-03161-5)
Supplement: Supplementary file 1 [file 10147_2026_3161_MOESM1_ESM.docx]

# **Supplementary Information accompanying**

**Clinical significance of WES-defined *ERBB2* amplification in resectable colorectal cancer**

Kazuki Kishi [1], Atsushi Hamabe [1], Yusuke Suwa [2], Naoya Akazawa [3], Keiji Hirata [4], Masataka Ikeda [5], Mitsuru Yokota [6], Kentaro Kato [7], Masahito Kotaka [8], Yujiro Nishizawa [9], Hideaki Bando [10], Yoshiaki Nakamura [10], Saori Mishima [10], Tadayoshi Hashimoto [10], Arkarachai Fungtammasan [11], Charuta C. Palsuledesai [11], Robert W. Lentz [11], Daisuke Kotani [10], Hiroya Taniguchi [12], Jun Watanabe [13], Ichiro Takemasa [14], Takeshi Kato [15], Mamoru Uemura [1], Hidetoshi Eguchi [1], Yuichiro Doki [1], Eiji Oki [16], Takayuki Yoshino [10]

**Affiliations**

[1] Department of Gastroenterological Surgery, Graduate School of Medicine, The University of Osaka, Suita, Japan.

[2] Department of Surgery, Gastroenterological Center, Yokohama City University Medical Center, Yokohama, Japan.

[3] Department of Gastroenterological Surgery, Sendai City Medical Center, Sendai Open Hospital, Sendai, Japan.

[4] Department of Surgery 1, School of Medicine, University of Occupational and Environmental Health, Kitakyushu, Japan.

[5] Division of Colorectal Surgery, Department of Gastrointestinal Surgery, Hyogo Medical University, Hyogo, Japan.

[6] Department of General Surgery, Kurashiki Central Hospital, Okayama, Japan.

[7] Department of Surgery, Teine-Keijinkai Hospital, Sapporo, Japan.

[8] Gastrointestinal Cancer Center, Sano Hospital, Kobe, Japan.

[9] Department of Gastroenterological Surgery, Osaka General Medical Center, Osaka, Japan.

[10] Department of Gastrointestinal Oncology, National Cancer Center Hospital East, Kashiwa, Japan.

[11] Natera, Inc., Austin, Texas, USA.

[12] Department of Clinical Oncology, Aichi Cancer Center Hospital, Nagoya, Japan.

[13] Department of Colorectal Surgery, Kansai Medical University, Hirakata, Japan.

[14] Department of Gastroenterological Surgery, Osaka International Medical and Science Center, Osaka Keisatsu Hospital, Osaka, Japan

[15] Department of Colorectal Surgery, National Hospital Organization Osaka National Hospital, Osaka, Japan.

[16] Department of Advanced Medicine and Innovative Technology, Kyushu University Hospital, Fukuoka, Japan.

# **Corresponding author**

Atsushi Hamabe

Department of Gastroenterological Surgery, Graduate School of Medicine, The University of Osaka, Suita, Japan.

E-mail: ahamabe@gesurg.med.osaka-u.ac.jp

**Supplementary Tables**

**Supplementary Table 1**. Numbers of patients and DFS events excluded before the landmark analysis, overall and according to WES-defined *ERBB2* amplification status.

| **Figure** | **Before landmark**  **n** | **Excluded n (%)** | **Excluded with event**  **n (%)** | ***ERBB2* amp (+): excluded / total** | ***ERBB2* amp (-): excluded / total** | **P value** | ***ERBB2* amp (+):  events among excluded** | ***ERBB2* amp (-): events among excluded** | **P value** |
| --- | --- | --- | --- | --- | --- | --- | --- | --- | --- |
| Fig.3, Fig.S2 | 2531 | 44 (1.7%) | 39 (88.6%) | 3/40 (7.5%) | 41/2491 (1.6%) | 0.0310 | 3/3 (100.0%) | 36/41 (87.8%) | 1.0000 |
| Fig.4c | 1208 | 25 (2.1%) | 25 (100.0%) | 2/25 (8.0%) | 23/1183 (1.9%) | 0.0923 | 2/2 (100.0%) | 23/23 (100.0%) | 1.0000 |
| Fig.4d | 244 | 21 (8.6%) | 21 (100.0%) | 0/0 | 21/244 (8.6%) | 1.0000 | 0/0 | 21/21 (100.0%) | 1.0000 |
| Fig.4e | 1013 | 4 (0.4%) | 4 (100.0%) | 0/0 | 4/1013 (0.4%) | 1.0000 | 0/0 | 4/4 (100.0%) | 1.0000 |
| Fig.5b | 43 | 3 (7.0%) | 3 (100.0%) | 3/43 (7.0%) | 0/0 | 1.0000 | 3/3 (100.0%) | 0/0 | 1.0000 |
| Fig.S3a | 1183 | 23 (1.9%) | 23 (100.0%) | 0/0 | 23/1183 (1.9%) | 1.0000 | 0/0 | 23/23 (100.0%) | 1.0000 |
| Fig.S3b | 1257 | 25 (2.0%) | 25 (100.0%) | 0/0 | 25/1257 (2.0%) | 1.0000 | 0/0 | 25/25 (100.0%) | 1.0000 |
| Fig.S3c | 26 | 2 (7.7%) | 2 (100.0%) | 2/26 (7.7%) | 0/0 | 1.0000 | 2/2 (100.0%) | 0/0 | 1.0000 |
| Fig.S4b | 26 | 2 (7.7%) | 2 (100.0%) | 2/26 (7.7%) | 0/0 | 1.0000 | 2/2 (100.0%) | 0/0 | 1.0000 |

**Supplementary Table 2**. Recurrence sites in WES-defined ERBB2 amplified and non-amplified groups. The column titled “with other sites” indicates the number of patients with a concurrent recurrence at site(s) other than the one indicated by the corresponding row.

|  | **WES-defined *ERBB2* amp(+) (N = 12)** | | **WES-defined *ERBB2* amp(-) (N = 520)** | |
| --- | --- | --- | --- | --- |
| **Characteristic** | **patients**^1^ | **with other sites**^1^ | **patients**^1^ | **with other sites**^1^ |
| **Recurrence Site** |  |  |  |  |
| *Liver* | 5 (42%) | 0 | 169 (27%) | 40 |
| *Lung* | 2 (17%) | 0 | 148 (24%) | 32 |
| *Peritoneum* | 2 (17%) | 0 | 109 (17%) | 46 |
| *Lymph node* | 1 (8.3%) | 0 | 62 (9.9%) | 35 |
| *Local* | 0 (0%) | 0 | 22 (3.5%) | 8 |
| *Ovary* | 0 (0%) | 0 | 11 (1.8%) | 7 |
| *Bone* | 0 (0%) | 0 | 8 (1.3%) | 4 |
| *Spleen* | 0 (0%) | 0 | 4 (0.6%) | 2 |
| *Brain* | 0 (0%) | 0 | 3 (0.5%) | 1 |
| *In the pelvis* | 0 (0%) | 0 | 2 (0.3%) | 1 |
| *Abdominal wall* | 0 (0%) | 0 | 1 (0.2%) | 1 |
| *Anal canal* | 0 (0%) | 0 | 1 (0.2%) | 0 |
| *Anterior part of the sacrum* | 0 (0%) | 0 | 1 (0.2%) | 0 |
| *Craniocervical part* | 0 (0%) | 0 | 1 (0.2%) | 0 |
| *Iliac muscle* | 0 (0%) | 0 | 1 (0.2%) | 1 |
| *Internal iliac artery* | 0 (0%) | 0 | 1 (0.2%) | 1 |
| *Kidney* | 0 (0%) | 0 | 1 (0.2%) | 1 |
| *Mesentery around the anastomosis* | 0 (0%) | 0 | 1 (0.2%) | 1 |
| *Pancreas* | 0 (0%) | 0 | 1 (0.2%) | 0 |
| *Pelvic plexus* | 0 (0%) | 0 | 1 (0.2%) | 1 |
| *Pleura* | 0 (0%) | 0 | 1 (0.2%) | 1 |
| *Port site* | 0 (0%) | 0 | 1 (0.2%) | 1 |
| *Pubis* | 0 (0%) | 0 | 1 (0.2%) | 0 |
| *Right quadratus lumborum muscle* | 0 (0%) | 0 | 1 (0.2%) | 1 |
| *Sacrum* | 0 (0%) | 0 | 1 (0.2%) | 1 |
| *Skin* | 0 (0%) | 0 | 1 (0.2%) | 1 |
| *Small intestine* | 0 (0%) | 0 | 1 (0.2%) | 0 |
| *Stomach* | 0 (0%) | 0 | 1 (0.2%) | 0 |
| *Unknown* | 2 (17%) | 0 | 69 (11%) | 0 |
| ^1^n (%) | | | | |

**Supplementary Table 3**. Baseline demographic and clinical characteristics of pStage III WES-defined ERBB2 amplified patients stratified by ACT receipt.

| **Characteristic** |  | **No ACT (n=421)** | **ACT (n=1070)** | **P value**  **(Fisher or Wilcoxon)** |
| --- | --- | --- | --- | --- |
| Sex | Male | 227 (53.9%) | 561 (52.4%) | 0.6448 |
|  | Female | 194 (46.1%) | 509 (47.6%) |  |
| Age, years | Median (IQR) | 71 (62-77) | 66 (55-73) | <0.0001 |
| Primary tumor location | Right (C-T) | 143 (34.0%) | 363 (33.9%) | 0.7500 |
|  | Left (D-RS) | 211 (50.1%) | 520 (48.6%) |  |
|  | Rectum (Ra, Rb) | 67 (15.9%) | 187 (17.5%) |  |
| Histological type | Differentiated | 403 (95.7%) | 993 (92.8%) | 0.3993 |
|  | Poorly differentiated/ Signet-ring cell | 15 (3.6%) | 50 (4.7%) |  |
|  | Other/Unknown | 3 (0.7%) | 27 (2.5%) |  |
| RAS mutations | Wild type | 234 (55.6%) | 576 (53.8%) | 0.5172 |
|  | Mutant | 173 (41.1%) | 461 (43.1%) |  |
|  | Unknown | 14 (3.3%) | 33 (3.1%) |  |
| BRAF V600E mutation | No | 373 (88.6%) | 945 (88.3%) | 0.9170 |
|  | Yes | 34 (8.1%) | 89 (8.3%) |  |
|  | Unknown | 14 (3.3%) | 36 (3.4%) |  |
| MSI | MSI-high | 39 (9.3%) | 75 (7.0%) | 0.1618 |
|  | MSS | 366 (86.9%) | 938 (87.7%) |  |
|  | Unknown | 16 (3.8%) | 57 (5.3%) |  |
| Early post-operative MRD status | Negative | 331 (78.6%) | 682 (63.7%) | 0.0069 |
|  | Positive | 58 (13.8%) | 186 (17.4%) |  |
|  | Unknown | 32 (7.6%) | 202 (18.9%) |  |
| Abbreviations: IQR, interquartile range; Right (C-T), cecum to transverse colon (proximal colon); Left (D-RS), descending colon to Rectosigmoid (distal colon); Ra, upper rectum; Rb, lower rectum; MSI, microsatellite instability; MSS, microsatellite stable; MRD, molecular residual disease. P-value was calculated using Fisher’s exact test among non-missing categories. | | | | |

**Supplementary Table 4**. Baseline demographic and clinical characteristics of pStage III WES-defined ERBB2 amplified patients stratified by ACT receipt.

| **Characteristic** |  | **No ACT (n=7)** | **ACT (n=22)** | **P value**  **(Fisher or Wilcoxon)** |
| --- | --- | --- | --- | --- |
| Sex | Male | 6 (85.7%) | 11 (50.0%) | 0.1872 |
|  | Female | 1 (14.3%) | 11 (50.0%) |  |
| Age, years | Median (IQR) | 66 (53-68) | 66 (61-73) | 0.2018 |
| Primary tumor location | Right (C-T) | 2 (28.6%) | 3 (13.6%) | 0.5259 |
|  | Left (D-RS) | 4 (57.1%) | 12 (54.5%) |  |
|  | Rectum (Ra, Rb) | 1 (14.3%) | 7 (31.8%) |  |
| Histological type | Differentiated | 7 (100.0%) | 21 (95.5%) | Not estimable |
|  | Other/Unknown | 0 (0.0%) | 1 (4.5%) |  |
| RAS mutations | Wild type | 7 (100.0%) | 20 (90.9%) | 1.0000 |
|  | Mutant | 0 (0.0%) | 2 (9.1%) |  |
| BRAF V600E mutation | No | 7 (100.0%) | 22 (100.0%) | Not estimable |
|  | Yes | 0 (0.0%) | 0 (0.0%) |  |
| MSI | MSI-high | 0 (0.0%) | 0 (0.0%) | Not estimable |
|  | MSS | 7 (100.0%) | 22 (100.0%) |  |
| Early post-operative MRD status | Negative | 6 (85.7%) | 14 (63.6%) | 1.0000 |
|  | Positive | 1 (14.3%) | 5 (22.7%) |  |
|  | Unknown | 0 (0.0%) | 3 (13.6%) |  |
| Abbreviations: IQR, interquartile range; Right (C-T), cecum to transverse colon (proximal colon); Left (D-RS), descending colon to Rectosigmoid (distal colon); Ra, upper rectum; Rb, lower rectum; MSI, microsatellite instability; MSS, microsatellite stable; MRD, molecular residual disease. P-value was calculated using Fisher’s exact test among non-missing categories. | | | | |

**Supplementary Table 5**. The distribution of ACT regimens and treatment durations in pStage III patients.

| **Regimen type** | **≤2 months** | **≤4 months** | **>4 months** | **Unknown** |
| --- | --- | --- | --- | --- |
| Fluoropyrimidine+Oxaliplatin | 80 | 352 | 540 | 7 |
| Fluoropyrimidine-only | 9 | 13 | 80 | 0 |
| Other / Unknown | 1 | 2 | 1 | 7 |

**Supplementary Table 6**. The distribution of ACT regimens and treatment durations in pStage III WES-defined *ERBB2* amplified and non-amplified groups.

| **WES-defined *ERBB2* amplification** | **Regimen type** | **≤2 months** | **≤4 months** | **>4 months** | **Unknown** |
| --- | --- | --- | --- | --- | --- |
| No | Fluoropyrimidine + Oxaliplatin | 77 | 345 | 530 | 7 |
|  | Fluoropyrimidine only | 9 | 13 | 79 | 0 |
|  | Other / Unknown | 1 | 2 | 1 | 6 |
| Yes | Fluoropyrimidine + Oxaliplatin | 3 | 7 | 10 | 0 |
|  | Fluoropyrimidine only | 0 | 0 | 1 | 0 |
|  | Other / Unknown | 0 | 0 | 0 | 1 |

**Supplementary Figures**

**Fig. S1**. Distribution of *ERBB2* copy numbers among individual patients. For patients with no *ERBB2* copy number variants, the copy number value was set to 2.0.

**Fig. S2**. Multivariable analysis of DFS excluding ctDNA-based MRD status among patients with complete clinicopathologic data available (N=2,487). Landmark analysis was performed.

**Fig. S3.** Multivariable analyses of DFS according to WES-defined *ERBB2* amplification status among pStage III patients with complete clinicopathologic data available. Multivariable Cox proportional hazards analysis in patients with WES-defined *ERBB2*-non-amplified colorectal cancer (N=1,160) (a), in patients with WES-defined *ERBB2*-non-amplified colorectal cancer adjusted for age, ACT, and early post-operative ctDNA-based MRD status during 2-10 weeks post-surgery (N=1,232) (b), and in patients with WES-defined *ERBB2*-amplified colorectal cancer adjusted for age, ACT, and early post-operative MRD status (N=24) (c). Landmark analysis was performed for all panels.

**Fig. S4**. **Prognostic significance and association between *ERBB2* copy number and early post-operative MRD status (2-10 weeks post-surgery) in patients with pStage III, WES-defined *ERBB2* amplified CRC**. (a) ERBB2 copy number stratified by recurrence status in patients with WES-defined *ERBB2* amplification and pStage III (N = 29). (b) Kaplan–Meier curve for DFS stratified by early post-operative MRD status in patients with WES-defined *ERBB2* amplification and pStage III (N = 24).

**Fig. S5 Sensitivity analysis using *ERBB2* amplification defined as *ERBB2* copy number between 3-20.** A) univariate association of WES-defined *ERBB2* amplification with DFS; B) multivariable analysis of DFS among patients with complete clinicopathologic data available; C) efficacy of ACT for pStage III patients without WES-defined *ERBB2* amplification; D) efficacy of ACT for pStage III patients with WES-defined *ERBB2* amplification; E) efficacy of ACT for pStage III patients without WES-defined *ERBB2* amplification with early post-operative MRD-positivity; F) efficacy of ACT for pStage III patients without WES-defined *ERBB2* amplification with early post-operative MRD-negativity.
